# Ethical considerations in the design, execution, and analysis of clinical trials of chronic pain treatments

**DOI:** 10.1097/PR9.0000000000000646

**Published:** 2018-03-29

**Authors:** Michael C. Rowbotham, Michael P. McDermott

**Affiliations:** a California Pacific Medical Center Research Institute, San Francisco, CA, USA; b Center for Human Experimental Therapeutics, University of Rochester Medical Center, Rochester, NY, USA

**Keywords:** Ethics, Clinical trials, Transparency in research, Belmont Report

## Abstract

**Introduction:**

In the field of pain research, clinical trials may randomize over 500 subjects and include more than 150 sites spanning over a dozen countries.

**Methods:**

This review examines the ethical considerations affecting clinical trial design, execution, and analysis of trials for chronic pain. The Belmont Report has been the touchstone for human studies protection efforts since 1979. Commissioned by the U.S. government in response to ethical failures in medical research, such as the Tuskegee Syphilis Study, the report emphasizes 3 basic principles: respect for persons, beneficence, and justice. Trial design and sample size have important ethical implications.

**Conclusions:**

Measures to enhance trial transparency and combat publication and many other types of bias should be implemented.

## 1. Introduction

An increasing proportion of clinical trials are international endeavors.^21^ A single registration trial may enroll thousands of subjects. Even clinical studies of approved compounds for pain may randomize over 500 subjects at over 150 sites in over a dozen countries.^4^ An entire industry of private, independent clinical research organizations has emerged to help trial sponsors determine as quickly as possible if an experimental compound, device, or treatment strategy is effective and safe. This review examines the ethical considerations affecting design, execution, and analysis of trials for chronic pain. Although centered on the U.S. system and phase 2 trials, the issues and recommendations apply internationally and to all types of clinical trials.

## 2. The Belmont Report

Commissioned by the U.S. Government in response to ethical failures in medical research, such as the Tuskegee Syphilis Study,^38^ legislation approved in July 1974 created the National Commission for the Protection of Human Subjects of Biomedical and Behavioral Research. After lengthy deliberations, the Belmont Report was published in 1979.^27^ At just under 5000 words, the Belmont Report has been the touchstone for protection of human subjects ever since. The recommendations contained in the Belmont Report are guided by 3 basic principles: respect for persons, beneficence, and justice (Table 1).

**Table 1:**
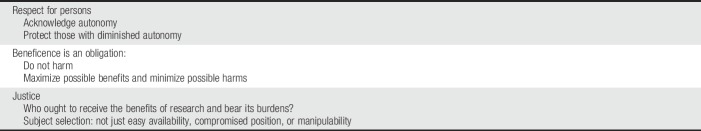
Institutional review board review and informed consent guidance from the Belmont Report of 1979.

Respect for persons incorporates 2 ethical convictions: that individuals should be treated as autonomous agents, and that persons with diminished autonomy are entitled to protection. An autonomous person is capable of deliberating about personal goals and acting under the direction of such deliberation. Withholding information necessary to make a considered judgment, when there are no compelling reasons to do so, demonstrates a lack of respect. Subjects, to the degree that they are capable, must be given the opportunity to choose what shall or shall not happen to them. Subjects not capable of autonomy must be protected.

Beneficence in the Belmont Report is understood not as an act of charity but as an obligation. The Hippocratic maxim “do no harm,” when extended to the realm of research, means that one should not injure 1 person, regardless of the benefits that might come to others. However, learning what will be of benefit may require exposing persons to risk. When is research justifiable, despite the risks involved?

The justice issue can be summarized through this question: Who ought to receive the benefits of research and who ought to bear its burdens? During the 19th and early 20th centuries, most research subjects in the United States were patients too poor to have other options for obtaining treatment, a charge still sometimes leveled against research conducted in impoverished countries, where the local population could never afford the treatments being tested were they to receive regulatory approval.^37^ Subjects must be selected for reasons directly related to the problem being studied.

A research consent is valid only if voluntarily given. Coercion occurs when an overt threat of harm is intentionally presented, and may be as simple as threatening to end the patient–physician relationship. Patients are often fearful of losing this relationship, and a patient's belief, even if unwarranted, that displeasing the physician–investigator might adversely affect care can be enough to induce consenting to research. Descriptions of research benefits (or minimization of risk) that are excessive, unwarranted, or inappropriate are usually detected by institutional review board members, as are improper rewards or inducements to participate. Undue influence can be subtle. Patients with treatment-refractory chronic pain often meet the definition of a vulnerable population, that is, having diminished capacity to consent, or willingness to accept very high risks in their search for a cure (Table 2).

**Table 2:**
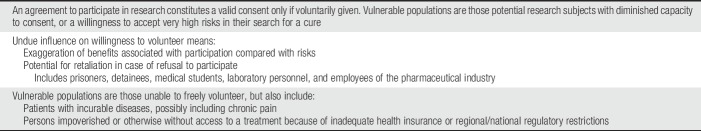
Vulnerable (“special”) populations.

## 3. Institutional review board review and informed consent

Institutional review board committee reviews protect the rights and welfare of human subjects by ensuring that proposed research study design and methods are scientifically sound and properly balance risks and potential benefits. Institutional review boards are empowered to approve (or reject), monitor, and regularly review research involving humans. The U.S. Office for Human Research Protections provides decision charts on how to apply the regulations.^20^

As guided by the Belmont Report, the consent process itself spans 3 elements: information, comprehension, and voluntariness. Much information is routinely required, such as the purposes and risks and anticipated benefits of the research procedure(s), alternatives to participation, and a statement offering the subject the opportunity to ask questions and to withdraw at any time from the research. Institutional review boards must decide how much information should be presented; an especially difficult task when experimental treatment is embedded in complex regimens carrying significant risk but are approved, or even Standard of Care. Describing alternatives to research participation and presenting only those studies appropriate and consonant with a potential subject's interests and wishes can be very time-consuming for investigators.

Comprehension is particularly important. Institutional review boards are good at determining if consent form language is at too high a level (beyond high school graduate or 1–2 years of college) to be readily comprehended by patients. Investigators are concerned with consent form length, which may exceed 10 pages for a simple minimal risk study. Understanding improves if potential subjects have several electronic device or online options to hear, view, or read the consent form at their own pace.^17,18,32^ Evidence indicates that participants do not carefully read through paper consents or e-consents before signing. Interactive online testing with immediate feedback and verifying comprehension before the subject can sign the consent form may be sufficient for low-risk studies, but direct interaction between investigator and subject persists.

## 4. Equipoise and the choice of comparison treatments in randomized trials for chronic pain

Clinical equipoise, a term first used by Freedman in 1987, is present “if there is genuine uncertainty within the expert medical community—not necessarily on the part of the individual investigator—about the preferred treatment.”^13^ For a typical randomized, controlled registration study evaluating a new drug, the issue of equipoise comes up primarily in the choice of subjects.^8–10,34^ Meticulous longitudinal studies show that for a patient newly diagnosed with epilepsy, the likelihood of success (defined as achieving a seizure-free state) steadily declines with each new therapy tried. In a study of 470 newly diagnosed epilepsy patients treated sequentially with a randomized choice of monotherapy using conventional antiepileptic drugs (AEDs), the first drug achieved seizure-free status in 47%.^23^ In those who failed to achieve a seizure-free state, the second AED increased the total seizure-free percentage by another 13%. The third AED tried as monotherapy added only another 1% to the total seizure-free proportion. Duotherapy added another 3% to the seizure-free group. At the end, 36% still had uncontrolled seizures. A subsequent study in an even larger group included numerous new AEDs but produced similar findings.^2^ Independent of what drugs have already been tried, by the third or fourth round of therapy, the chances of complete seizure control success in the treatment refractory are very small indeed.

For pain, the epilepsy trials hypothesis has not been as rigorously tested. It would be helpful if more information from deeper data analyses of previously conducted clinical trials were available. Were the analogy to hold true, sponsors should prefer to enroll patients with as few unsuccessful previous treatment attempts as possible (Table 3). Academic center pain clinics are disadvantaged by their preponderance of heavily treated, medically complex, and thoroughly treatment-refractory patients. The ethical issue arises because equipoise may not be present at the level of the individual patient. Here is the conundrum: given a choice between a time-tested, clinically available treatment that has a specific regulatory approval for the pain disorder being treated and an experimental compound with unproven safety and efficacy in a phase 2 proof-of-concept trial, what is the ethical decision? The Hippocratic maxim of “do no harm,” as well as the expectation that patients will be provided with treatment that meets the local standard of care, clearly favors trying all approved, efficacious treatments before enrolling a patient in the phase 2a trial. Yet, this action could doom the study to failure by having its participants be thoroughly treatment refractory and therefore much less likely to respond than that the literature would suggest. For a patient with stable and nonprogressive pain for whom foregoing an approved treatment would not result in additional risk of serious or irreversible harm, the ethical dilemma may be softened. To further complicate the matter, the screening process before randomization may not thoroughly document all previous treatments (dose, duration, response, etc). This information is hard to obtain from the medical record and patients may not remember or be reluctant to disclose information they feel could jeopardize their chance of entering the trial.

**Table 3:**
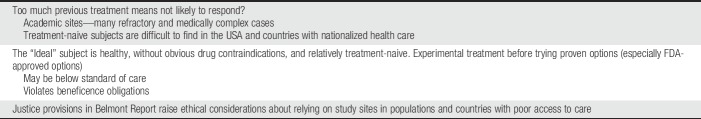
Ethical considerations in pain trials.

What if the comparison treatment(s) in a study are approved therapies? This strategy provides a reference point of response to standard treatment. Subjects already unsuccessfully treated with multiple accepted therapies other than the study comparison treatment may be less likely than relatively treatment-naive subjects to respond to either experimental treatment or comparison treatment. The ethical issue described in the preceding paragraphs is not fully resolved, however, and devising inclusion/exclusion criteria is difficult.^8–10,15,34^ If potential subjects have already been exposed to one of the comparison therapies, trying the exact same drug again under blinded conditions is hard to justify. That may not hold for drugs that share the same mechanisms of action. For example, previous cross-over studies show that the closely related drugs amitriptyline and nortriptyline may produce divergent responses in the same patient.^36^ A strategy randomizing to only those accepted therapy arms that the patient has not tried better mitigates the issue. Studies with conventional therapy as comparator arms balance the benefit of access to accepted therapies against the risk of exposure to an unproven experimental treatment.

## 5. Placebo controls and strategies to manage the placebo response

The word placebo translates from Latin as “I shall please.” Defined as an “inactive substance,” patients in placebo arms, nonetheless, can show substantial reductions in pain. The updated 2013 Helsinki Declaration outlines when placebo controls are justified.^39^ How and when to incorporate a placebo or sham treatment and interpret the results is complex and fraught with ethical implications.

If the goal of a trial is to determine efficacy, randomization with placebo controls is a highly rigorous trial design option, but there are many alternatives to randomized clinical trials (RCTs).^15^ The ethical problem with using placebo treatment as a control is that 1 group of patients will have their chronic pain go untreated. As far back as the 1950s, “active placebo” drugs (such as dicyclomine, clonidine, benztropine, and lorazepam) have been used to improve blinding by producing side effects similar to the study drug, but they are not without their own risks.^10,16,34^ When the trial protocol requires discontinuation or tapering of analgesic medications before starting study treatment, patients assigned to placebo may experience worsened pain throughout the study (unlike those assigned to study drug if the drug proves effective). Sponsors often exclude trial subjects from participating in subsequent trials. There is no ethical obligation to guarantee exposure to the experimental treatment; after all, the trial is presumably being performed because of uncertainty about whether or not the experimental treatment is efficacious and reasonably safe.

Liberal use of rescue medications in placebo-controlled trials muddies the waters further. Patients in the placebo group may derive enough pain reduction from the rescue medication (especially if opioids are used as “rescue”) to eliminate any differentiation from the experimental treatment, in which case the study could end up with falsely negative results. Conversely, if the “rescue” medication is completely ineffective, then it is not a rescue!

One strategy that is sometimes considered is to identify early in a study the “placebo responders” and eliminate them from the trial.^29^ Starting with a single-blind placebo run-in period means the study staff is unblinded and they may bias or shape the patient's response. It also wastes the subject's time. Furthermore, who is a “placebo responder” and how is he/she identified? There is no agreed on criterion; early termination of such a subject would be ethically dubious. Any analgesic response short of becoming completely pain-free during initial placebo treatment leaves the possibility that the response to active treatment might be greater or more enduring.

Trials of surgical therapies and implanted devices can use sham procedures as a control.^1,14,24,26^ Because the control therapy cannot be blinded at the level of the surgeon/interventionalist, efficacy measures are collected by independent raters. In some cases, the sham procedure(s) can be complex. In a trial of tissue implantation for Parkinson disease, sham surgery included the fitting of a stereotactic frame on the subject, the administration of anaesthetic, drilling of partial burr holes in the skull that did not penetrate the dura, positron emission tomography scans, and treatment with antibiotic and immunosuppressive drugs.^28,29^ The ethical disadvantage of enrolling a subject and exposing them to the risks of sham therapy is counterbalanced by the importance of methodological rigor in evaluating the efficacy and safety of the experimental procedure. Particularly, in the setting of a chronic pain condition for which outcome measures are derived through self-report and responses are inherently subjective, blinding to treatment assumes even greater importance in study design. In addition, placebo effects can be greater in trials of more invasive interventions,^33^ causing participants receiving an ineffective invasive intervention to exhibit better responses. Alternative study designs such as the use of historical controls or randomization to a best available medical therapy control group may be prone to biases that overestimate the effects of the invasive intervention. Given the costs and risks associated with invasive treatments, it is imperative to avoid falsely declaring a procedure to be effective and allowing it to be adopted in practice. Of course, the risks associated with the sham procedure would have to be carefully weighed against the potential benefits of a more rigorous study design in any individual study.

If a placebo is a “negative” control, a known effective treatment arm can be included as a “positive” control with the goal of further increasing the assay sensitivity of the trial. This design maneuver adds to study cost and the total number of subjects needed, and does not avoid exposure to placebo in cases that present an ethical dilemma.^8–10,34^ Enriched enrollment randomized withdrawal designs guarantee that all subjects will be exposed to the study compound under study. Only trial-defined “responders” progress to randomized blinded treatment, raising the ethical question of whether “responders” adequately represent the actual clinical population.^22^ Although the design feature of access to the study intervention may make it easier for subject recruitment, this by itself does not resolve any ethical issues given that the safety and efficacy of the intervention would not have been established. Such designs typically minimize exposure to placebo, particularly if an endpoint such as time to require rescue medication is incorporated. These designs can be efficient in terms of number of subjects needed. However, there is the implicit assumption that pain increase during withdrawal of an effective medication (through crossing over to placebo) is equivalent to initial pain reduction.

Placebo response is not a stable property and varies by condition. A trials data set that merged results across registration trials of several different drugs showed that placebo response magnitude tended to increase over time. Some studies that show superiority of the experimental treatment in the initial weeks of treatment end up as negative trials when the placebo group gradually “catches up” at the end of a 12 week or longer treatment period.^30^ Placebo-controlled trials in painful diabetic neuropathy show an average pain reduction of 26% with placebo, but only 15% in studies of postherpetic neuralgia. Furthermore, 1 event-triggered multiple exposure trial showed relatively stable responses to active drug with each exposure but steadily declining responses to placebo.^11^ By the fourth cycle, most subjects in the placebo group had dropped out and only 11% of those remaining reported pain relief (compared with 88% assigned to active treatment).

## 6. Ethical aspects of sample size in clinical trials

Clinical trials with too few subjects run the risk of failing to find an effect when one exists. Conversely, given that a trial finds an effect, the probability that the treatment is truly effective is smaller with a smaller study than it is with a larger one.^3,12^ Exposing as few subjects as possible to the risks of an experimental treatment has ethical advantages, but discarding beneficial therapies based on an underpowered study is ethically unsound, as is having ineffective therapies be accepted. Conducting additional studies to try to verify the results of an underpowered but positive study may lead to even more patients being exposed to the risks of the study treatment than if the original study were larger.

Studies with active comparators expose an additional group of patients to the risks of the active comparator and should require more subjects to demonstrate superiority than when the comparator is a placebo. Phase 2 studies with a new compound should probably use placebo as a control because the goal is determine if any preliminary evidence of efficacy is present. Later studies, when much more safety data are available, can be larger and include an active comparator because one of the goals of the study may be to determine if the study drug represents a true advance over current therapy, ie, a “clinically significant” benefit.

## 7. Randomization risk and time risk

Randomization risk refers to patients assigned to placebo or sham treatment.^25^ They are not expected to experience meaningful benefit, yet they devote their time and energy to study participation. Other options, such as entering a different study, and perhaps being assigned to active treatment with a drug that proves efficacious, would in retrospect have been the better choice. Some might not view this as an important risk, for example, in patients with stable nondisabling chronic pain, such as postherpetic neuralgia in a person 75 years of age. However, some studies have inclusion/exclusion criteria that limit access to patients within a defined period after disease onset. The potential subject could also have used the time to try nonstudy treatments through their regular physician.

## 8. Evidence-based medicine has feet of clay

Guidelines on therapy may combine a systematic review of the literature plus “expert opinion.” The best evidence comes from RCTs with placebo groups or other appropriate controls to minimize bias. The more “positive results” data from published RCTs, the stronger the recommendation in favor of a particular treatment. Conversely, the fewer negative results or articles about safety concerns, the stronger the favorable recommendation. Publication bias arises from the tendency for sponsors, researchers, and editors to handle experimental results that are positive differently from results that are negative or inconclusive.^6,25,31^ Publication bias in the form of nonpublication wastes the contributions of (and risks taken by) patients to advance medical knowledge.

Taking advantage of the many approvals of new antidepressants and the fact that the U.S. Food and Drug Administration (FDA) publishes its complete dossier on the fda.gov web site shortly after approval, Turner and colleagues published a landmark study in 2008 demonstrating the hazards of publication bias.^35^ In their words, “Not only were positive results more likely to be published, but studies that were not positive, in our opinion, were often published in a way that conveyed a positive outcome.” They calculated that the effect size of the still experimental antidepressants in the published literature overestimated their true effect size by ∼30%. They also found instances of selective outcome reporting in which primary measures were downgraded to secondary status, and secondary measures promoted. Publication bias and selective outcome reporting are ethical breaches, falsely raising the expectations of prescribing physicians and patients alike. There are implications for the design of future trials. Sample size calculations for a trial to investigate if a new compound is superior to the approved reference compound may be incorrect if earlier literature reports overestimated the effect of the reference compound (relative to placebo) in the pivotal trials.

Many other types of reporting bias^6,25^ exist, such as: multiple publication bias (the same completed trial published in multiple journals or multiple progress reports without citing earlier reports); combinations of location, language, and citation bias (negative trials in journals not indexed in PubMed or non-English, positive trials in high impact journals without citing the negative trials); and perhaps the most frequent of all, time lag bias. Time lag bias is easiest to understand as a marketing strategy. Positive trial publications are timed to build excitement surrounding widespread availability of a new drug. Negative trials are published after the drug has already found a niche in the marketplace, or when negative publicity might be least damaging. Only the most carefully constructed treatment guidelines will go deeper than the published literature to analyze the full data set for each compound in a class and then compare the compounds. Selective publication and time lag bias can be defeated by analyzing the FDA's data set at the time of new drug approval, but all subsequent studies will be susceptible to these biases unless additional indications are granted and new data sets appear on fda.gov.

## 9. Enhancing transparency in the pain literature—RReACT, RReMiT, and RReADS

In 1997, the FDA created the ClinicalTrials.gov (CTG) registry. In 2005, clinical trial registration became a precondition for publication in an ICMJE (International Committee of Medical Journal Editors) journal.^5^ In the pain literature, the RReACT, RReMiT, and RReADS databases took a snapshot and scorecard approach to clinical trials of drugs for chronic pain and migraine, and trials of analgesic devices.^7,19^ The snapshot was a database of all clinical trials on CTG and other trials registries and the scorecard was the proportion of completed clinical trials that had results publicly available through posting on a registry, through press release or other documents available through an internet search, or through a peer-reviewed journal publication. Examining almost 1000 trials across a spectrum of painful disorders (fibromyalgia, diabetic painful neuropathy, postherpetic neuralgia, migraine, complex regional pain syndrome, and central poststroke pain) and types of treatment revealed no single study characteristic that consistently predicted unavailability of results. At 12 months after study completion, results reporting remained below 60% for peer-reviewed publications.

## 10. Summary

Ethical considerations affect nearly every aspect of clinical trial design, analysis, and reporting. Some recommendations can easily be made (Table 4). To reduce inadvertent bias and minimize unnecessary within-subject and between-subject variability, trial subjects should be carefully trained on key outcome measures, including how to rate their pain. Incorporating an “active” placebo (such as an anticholinergic in a study of tricyclic antidepressants or a benzodiazepine in a study of a sedating compound) adds drug side-effect risks to the known risk of receiving an “inactive” therapy by being in the placebo group, and is therefore discouraged. Invasive methods to blind treatment assignment, such as sham surgery and sham implantation of a device, are justified in some cases because they can provide a definitive answer on benefit with fewer patients being exposed to a treatment compared with nonblinded studies using natural history controls or best medical care controls.

**Table 4:**
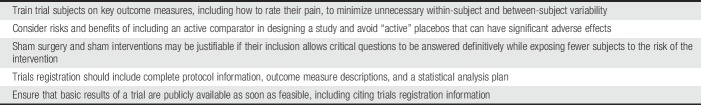
Recommendations.

In clinical trials of new drugs, use of placebo controls, blinded administration of a standard efficacious therapy as a study arm, and use of rescue medications have important ethical implications that affect inclusion/exclusion criteria and sample size calculation. “Ideal” populations in clinical trials may not adequately represent actual patient populations, potentially explaining some of the lack of translation of trial results to clinical practice. Potential subjects and investigators need to be aware of seemingly obscure risks such as time risk and randomization risk.

Measures to enhance trial transparency and combat publication bias should be implemented. Some are simple, like requiring more comprehensive descriptions of protocols (including outcome measures and statistical analysis plans) on trials registration databases (or publication as a full-length article), and citing the CTG registration number (enabling journal reviewers to comparing articles with registry entries). Additional recommendations to increase availability of trial results include enforcing existing results reporting regulations, enabling all primary registries to post results, and reducing barriers to publishing “negative” trials.^31^ For all diseases and treatment modalities, evidence-based medicine should explore ways to rigorously adjust for the sheer magnitude of missing results in formulating treatment recommendations. Ethical considerations should assume a more prominent role, especially in trial design.

## Disclosures

The authors have no conflict of interest to declare.

This work was made possible by generous gifts to the CPMC Foundation from multiple donors.
